# Electrospun Silk-Boron Nitride Nanofibers with Tunable Structure and Properties

**DOI:** 10.3390/polym12051093

**Published:** 2020-05-11

**Authors:** Ye Xue, Xiao Hu

**Affiliations:** 1 Department of Physics & Astronomy, Rowan University, Glassboro, NJ 08028, USA; xuey5@rowan.edu; 2 Department of Biomedical Engineering, Rowan University, Glassboro, NJ 08028, USA; 3 Department of Molecular and Cellular Biosciences, Rowan University, Glassboro, NJ 08028, USA

**Keywords:** silk fibroin, hexagonal boron nitride, thermal analysis, glass transition, composite nanofibers

## Abstract

In this study, hexagonal boron nitride (h-BN) nanosheets and *Bombyx mori* silk fibroin (SF) proteins were combined and electrospun into BNSF nanofibers with different ratios. It was found that the surface morphology and crosslinking density of the nanofibers can be tuned through the mixing ratios. Fourier transform infrared spectroscopy study showed that pure SF electrospun fibers were dominated by random coils and they gradually became α-helical structures with increasing h-BN nanosheet content, which indicates that the structure of the nanofiber material is tunable. Thermal stability of electrospun BNSF nanofibers were largely improved by the good thermal stability of BN, and the strong interactions between BN and SF molecules were revealed by temperature modulated differential scanning calorimetry (TMDSC). With the addition of BN, the boundary water content also decreased, which may be due to the high hydrophobicity of BN. These results indicate that silk-based BN composite nanofibers can be potentially used in biomedical fields or green environmental research.

## 1. Introduction

Electrospinning is a unique technique to stretch polymer droplets at high speed by the electrostatic force and then phase-separate polymer chains and solidified them into fibers [1,2,3]. The physical properties of the fibers—such as diameter, morphology, mechanical strength, crystallinity, and specific surface area—can be controlled by adjusting the parameters of electrospinning such as solution flow rate, voltage, different collection design, and environmental factors (temperature, humidity, etc.) [4]. Controlling the diameter of the fibers also has great effect on their physical properties. When the fiber diameter is relatively large, the specific surface area of the fiber will be small, the arrangement of polymer molecular chains may become less ordered, and more defects on the fiber surface could generally reduce the mechanical strength of the fiber [2,5]. When the diameter of the fiber is less than 1 µm, the mechanical properties of the nanofiber will be greatly improved since the surface defects of the fiber are reduced [6]. Electrospinning is an effective technique to produce nanofibers with tunable properties that has been used in biomedical science, environmental research, and clean energy in recent years.

Silk protein is a renewable natural material with good biocompatibility and degradability [7,8,9]. Natural silk fiber has excellent mechanical properties, it has extremely high tensile strength and excellent ductility. These excellent functions make it have good application prospects in the medical fields, such as tissue scaffolds, biosensors, and wound dressings [10,11,12]. Silk proteins usually come from silkworms and spiders. Different sources of silk proteins lead to different physical and chemical properties. Genetic engineering techniques have also been used to adjust gene sequences to manipulate amino acid composition to obtain silk protein materials with new functions [13,14,15]. The secondary structure of silk protein has also been well studied, and the structure of silk protein can be manipulated through various post-processing methods [16,17]. Natural silk proteins and recombinant silk proteins have been widely used as scaffolds for tissue engineering, as well as microspheres for drug delivery [18,19,20,21]. Many biosensors and bioelectronics also require temperature sensors or heat transfer components [22,23]. It was found that the thermal conductivity of silk can be improved by increasing the crystallinity of silk proteins, although it is still much lower than many inorganic materials [24,25,26]. On the other hand, boron nitride (BN) nanomaterials have excellent thermal stability, thermal conductivity, and stable chemical properties, and have long-term good insulation properties [27,28,29,30]. Cell and animal experiments have also shown that boron nitride is not cytotoxic in the proper dosage range and does not have any negative effects on animal signs, which indicates that boron nitride is also biocompatible [31,32,33]. In recent years, the application of boron nitride in the field of biomedicine has been developed. Functionally modified boron nitride nanoparticles have made significant progress as a tumor drug delivery system [34,35,36]. However, the hydrophobicity and extremely stable chemical properties of boron nitride nanosheets also limits its biomedical applications as a bulk material [35,37]. Therefore, combining hydrophobic boron nitride and hydrophilic silk protein into a composite material to have additional controllable functions will significantly expand the application range of the material.

In this study, h-BN nanosheets and *Bombyx mori* silk fibroin protein solutions were electrospun into nanofibers in various mixing ratios. The interaction between the h-BN and protein matrix were studied in detail. The impact of h-BN on the morphology and diameter of electrospun BN-silk fibroin (BNSF) fibers were investigated by scanning electron microscopy (SEM). The effect of h-BN on the secondary structure of silk protein was investigated using Fourier transform infrared spectroscopy (FTIR). Thermal stability and properties of the BNSF electrospun nanofibers were studied using thermogravimetric analysis (TGA) and temperature-modulated differential scanning calorimetry (TM-DSC).

## 2. Materials and Methods

### 2.1. Raw Materials

Bombyx mori silk cocoons were purchased from Treenway Silks (Lakewood, CO, USA). Silk cocoons were firstly degummed by boiling cocoon pieces in a 0.02 M NaHCO3 (Sigma-Aldrich, St. Louis, MO, USA) solution for 30 min. Then the degummed fibers were rinsed three times in DI water to thoroughly remove the sericin coatings. The rinsed silk fibroin fibers were dried in a vacuum oven at 25 °C overnight [38,39]. The following materials were used as purchased: formic acid (ACS Grade, 98%) was purchased from EMD Millipore Corporation (Burlington, MA, USA), calcium chloride (anhydrous, ACS Grade) was purchased from AMRESCO Inc. (Solon, OH, USA), and h-BN nanosheets were purchased from Sigma-Aldrich (St. Louis, MO, USA).

### 2.2. Material Synthesis

Dried silk fibroin (SF) fibers were dissolved in a formic acid solution with 4% w/v calcium chloride at a concentration of 0.15 g/mL. The SF solution was centrifuged to remove the undissolved residues at 5000 rpm for 10 min. h-BN was added into the solution at various weight ratios to have the BNSF blends with 5%, 10%, 20%, 30%, 40% of BN (For example, 5% suggests BNSF fibers consist of 95g silk protein and 5 g h-BN). The BNSF solution was shaken with a vortex mixer for 10 min. The thoroughly mixed BNSF solution was then electrospun into nanofibers at a voltage of 20 kV at room temperature and a relative humidity of about 50%. The solution flow rate was controlled at 20 µL/min using a syringe pump (Harvard Apparatus Model 22, Holliston, MA, USA). Electrospun samples were collected every 5 min between two parallel metal plates lined with aluminum foil, placed at 4 cm from the needle tip. The two parallel plates collecting design can help the solvent evaporate faster and slightly improve the alignment of the fibers as compared to that of the pad collector. In addition, free standing fiber mesh samples can be collected directly. The fibers were then dried in a vacuum oven at 40 °C for 24 h to remove the acid residues (verified by FTIR).

### 2.3. Surface Morphology Analysis

The electrospun fibers were characterized with a Leo 1530 VP scanning electron microscope (SEM) (Oberkochen, Germany). All samples were sputter-coated with gold before SEM imaging. Experiments were conducted with an accelerating voltage ranging between 10 and 20 kV.

### 2.4. Structure Analysis

Structure information of the electrospun fibers was obtained using a Bruker Tensor 27 Fourier transform infrared (FTIR) spectrometer (Billerica, MA, USA). The spectrometer is equipped with a deuterated triglycine sulfate detector, and a multiple reflection, horizontal MIRacle ATR attachment with a Ge crystal (Pike Tech, Madison, WI, USA). A continuously purging nitrogen gas was provided while the experiments were conducted. The spectra were taken at a range of 4000 to 400 cm^−1^ with 128 background scans and 128 sample scans at a resolution of 4 cm^−1^. Each sample was characterized at three different spots to ensure homogeneity.

### 2.5. Thermal Analysis

Thermogravimetric analysis (TGA) of BNSF nanofibers was investigated with a TA Instruments Q600 SDT instrument (Wilmington, DE, USA). Each sample weighed between 5–10 mg. Measurements were made from 25 °C to 600 °C at a heating rate of 10 °C/min. All experiments were conducted with continuous nitrogen gas flow rate of 50 mL/min.

Temperature modulated differential scanning calorimetry (TM-DSC) was conducted with a Q100 calorimeter (TA Instruments, Wilmington, DE, USA) equipped with a refrigerated cooling system. The N_2_ flow rate was set to 50 mL/min, and measurements started at −30 °C and ended at 400 °C. The temperature increased at a rate of 2 °C/min and was modulated every 60 s at an amplitude of 0.318 °C to measure the reversing heat capacity.

## 3. Results and Discussion

### 3.1. Morphology Study

**Figure 1:**
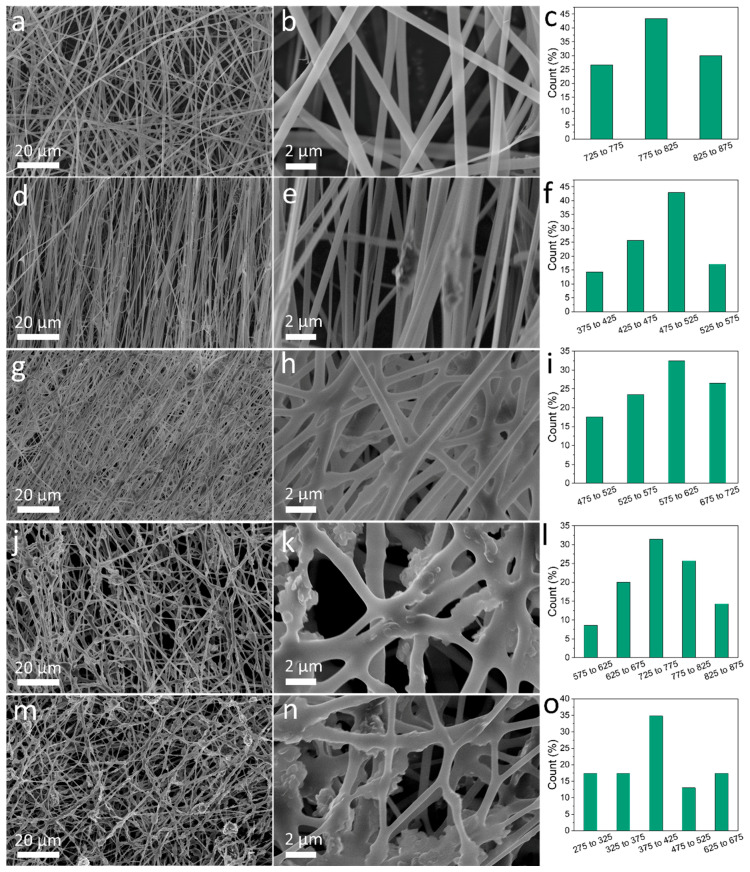
Scanning electron microscopy (SEM) images of pure silk fibroin (SF) (**a**,**b**), 5% boron nitride silk fibroin (BNSF) (**d**,**e**), 10% BNSF (**g**,**h**), 30% BNSF (**j**,**k**), and 40% BNSF (**m**,**n**) nanofibers; Quantitative analysis of diameter distribution of pure SF (**c**), 5% BNSF (**f**), 10% BNSF (**i**), 30% BNSF (**l**), and 40% BNSF (**o**) nanofibers.

SEM analysis was conducted to analyze the morphology and microstructure of the nanofibers (Figure 1). As seen in Figure 1a,b, pure SF nanofibers showed a smooth and uniform surface. For BNSF samples, h-BN nanosheets can be found either immersed into the fiber matrix or spread on the fiber surface. The 5% BNSF sample showed the best fiber alignment. When the concentration of h-BN is 10% or higher, it can be seen that the crosslinking density between the fibers is significantly increased (Figure 1e,h,k,n), suggesting the interactions between h-BN and silk fibroin molecules become stronger. The 30% and 40% BNSF samples have a much rougher surfaces due to the high content of h-BN nanosheets. The pure SF nanofibers had the largest fiber diameter with a size distribution centered around 800 nm, while all other samples showed smaller diameters with an average size of 400–750 nm.

### 3.2. Structural Study

**Figure 2:**
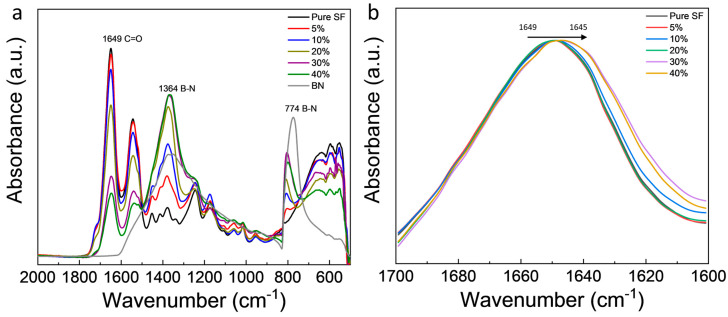
Fourier transform infrared spectroscopy (FTIR) spectra of (**a**) electrospun pure SF and BNSF nanofibers and raw BN sheets; (**b**) the amide I region of pure SF and BNSF nanofibers.

FTIR spectroscopy was used to characterize the interaction between h-BN and silk fibroin and the secondary structure of silk fibroin (Figure 2). All BNSF samples showed two peaks at 774 and 1364 cm^−1^, which were attributed to the h-BN sheets [40,41]. As the BN content increases, the intensity of these two peaks gradually increases. The raw BN sheets did not show absorbance in the amide I region (1600–1700 cm^−1^), which is mainly related to the secondary structure conformation of the protein. Both the pure SF sample and BNSF samples show a broad amide I peak, while the peak position of the samples shifted from 1649 cm^−1^ (pure SF) to 1645 cm^−1^ (40% BNSF). This indicates that the pure SF nanofibers are mainly composed of α-helical structure (centered around 1650 cm^−1^), and the high content of BN nanosheets in BNSF fibers can transform part of the secondary structures into random coils (centered around 1640 cm^−1^) [9,16]. During the dissolution and fiber drying process, the α-helix structures in the pure SF protein matrix may be disrupted by the BN nanosheets, and stronger hydrogen bonds were formed between the nitrogen atoms of the BN and the protein chains, which resulted in a reduction in the number of α-helixes in BNSF nanofibers [16].

### 3.3. Thermal Study

**Figure 3:**
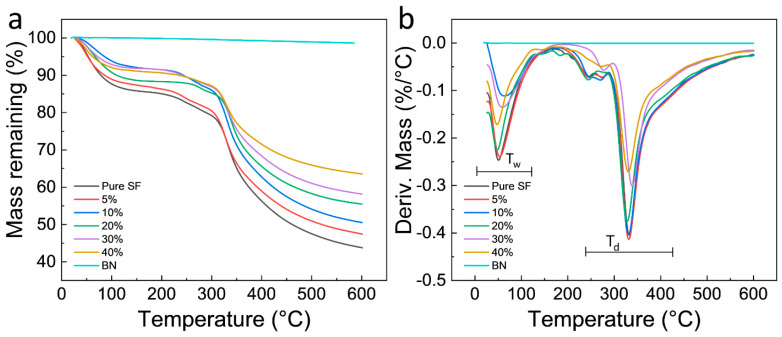
Thermogravimetric curves of (**a**) electrospun BNSF nanofibers and raw BN sheets; (**b**) displays the first derivative thermogravimetry (DTG) curves of the samples.

Thermal stability of BNSF nanofibers was first studied by TGA (Figure 3), and their thermal properties are summarized in Table 1. Pure BN was highly thermally stable with only 1.4% mass loss until 600 °C. In Figure 3, pure SF and BNSF electrospun nanofibers all showed a first mass loss step (*T*_w_–TGA) around 55 °C (Figure 3a), which is caused by the evaporation of bound water molecules. Silk protein can quickly absorb large amounts of water even in a low humidity environment. The pure SF showed the largest mass loss about 15% in this region, while the 40% BNSF sample showed the smallest mass loss about 7.4%. This difference can be attributed to the high hydrophobicity of BN sheets. All samples showed a major degradation temperature around 330 °C (*T*_d_–TGA, Figure 3b), which is attributed to the major decomposition of the silk proteins. There are two tiny mass loss steps shifting in the degradation region of 200–300 °C, which may be caused by the interactions between the silk protein and the BN. The residual mass of pure SF and BNSF electrospun fibers at 600 °C is between 43.7–63.6%, and the residual mass of electrospun fibers increases with the increase of BN content, which suggests that interactions between BN and SF improve the thermal stability of the fibers.

**Figure 4:**
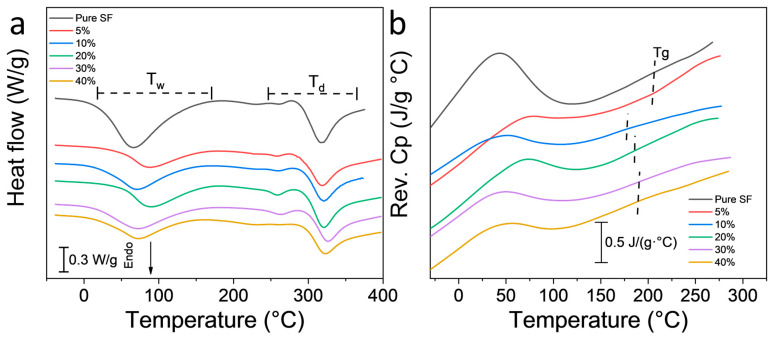
(**a**) Total heat flow curves of electrospun SF and BNSF nanofibers; (**b**) the reversing heat capacity curves of BNSF nanofibers. The scans were at a rate of 2 °C/min and temperature was modulated every 60 s at an amplitude of 0.318 °C.

Heat flow curves from TM-DSC scans (Figure 4a) confirmed the bounding water evaporation and protein decomposition temperatures from TGA. The peaks from bounding water evaporation (*T*_w_–DSC) are much broader than the protein decomposition peaks (*T*_d_–DSC), and the pure SF fibers showed the lowest T_w_ peak compared to all BNSF fibers, indicating the interaction between SF and BN molecules has significant impact on their bound water. Reversing heat capacity curves (Figure 4b) showed that pure SF has the highest onset glass transition temperature at about 120 °C, while 40% BNSF has the lowest onset glass transition temperature at about 96 °C. This suggests that amorphous components such as random coils in silk fibroin gained more mobility when combined with BN nanosheets. The thermal properties obtained from TM-DSC (water evaporation peak temperature, *T*_w_–DSC; glass transition temperature, *T*_g_–DSC; and thermal degradation peak temperature, *T*_d_–DSC) are also summarized in Table 1. The glass transition temperature of pure SF is higher than those of BNSF samples, indicating that the random coil structure in BNSF nanofibers can significantly improve the flexibility of the material.

### 3.4. Mechanism of Self-Assembly

**Figure 5:**
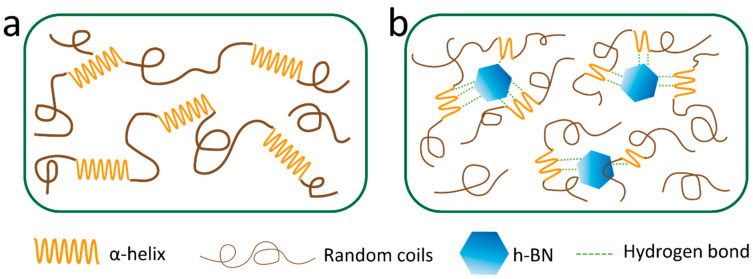
Self-assembled structures of (**a**) electrospun pure SF nanofibers; (**b**) electrospun BNSF nanofibers, showing that the BN nanosheets disrupted the secondary structures of silk fibroin proteins.

Based on the results of secondary structure analysis from FTIR and the thermal analysis from TGA and DSC, a mechanism of self-assembly for the BNSF nanofiber materials is proposed in Figure 5. As discussed in Figure 2, pure SF and BNSF samples showed a peak shift between 1645 and 1649 cm^−1^, which indicated that the electrospun pure silk fibers are rich in α-helical structures [16,42]. After adding the BN nanosheets, the peak shifted to a lower wavelength, which suggested that the α-helix structure in the protein matrix is reduced and the random coil structure is increased [16]. The hydrogen bonds between BN molecules and the protein backbones can effectively disrupt the α-helix structures and transform them into random coil structures (Figure 5b). Meanwhile, these hydrogen bonds between BN and SF proteins at different locations will also increase the numbers of crosslinks between the nanofibers, as shown in the SEM (Figure 1). The smaller and more dispersed distribution of α-helices resulted in higher mobility of the amorphous components, which in turn resulted in broader and lower glass transition temperatures of the composite nanofibers (Figure 4) [43].

## 4. Conclusions

In this study, the interactions between h-BN nanosheets and silk fibroin proteins in their electrospun nanofibers were investigated. The morphology and microstructure of the electrospun fibers were observed using SEM. Pure SF sample showed a smooth fiber surface with uniform fiber diameter. Electrospun fibers with a high BN content (over 10%) showed rougher surface morphology and higher fiber crosslinking density. FTIR results showed that BN molecules can form hydrogen bonds with silk proteins and transform the α-helical structure of pure SF into random coils, which suggests that the composite material has better flexibility. Due to the high hydrophobicity of BN and the strong interaction of SF-BN, pure SF samples showed the highest bound water content in TGA study and the lowest bound water evaporation temperature in DSC study. The good thermal stability of BN can significantly improve the thermal stability of electrospun BNSF fibers, and a self-assembly mechanism of electrospun BNSF nanofiber materials is proposed.

## Figures and Tables

**Table 1:** Thermal properties of pure silk fibroin (SF) nanofibers, different boron nitride silk fibroin (BNSF) nanofibers, and raw BN sheets.

Sample	Bound Water (%)-TGA	*T*_w_ (°C)-TGA	*T*_w_ (°C)-DSC	*T*_g_ (°C)-DSC	*T*_d_ (°C)-TGA	*T*_d_ (°C)-DSC	Mass Remaining% at 600 °C
**Pure SF**	13.6	50.8	66.2	204.9	332.2	319.0	43.7
**5% BNSF**	12.2	53.2	87.7	202.7	331.3	319.4	47.4
**10% BNSF**	8.5	57.9	71.3	177.9	331.6	321.3	50.5
**20% BNSF**	10.8	49.5	89.7	185.6	328.5	321.3	55.5
**30% BNSF**	8.4	54.2	74.4	189.5	339.1	327.2	58.2
**40% BNSF**	8.6	49.9	73.3	190.2	330.7	322.8	63.6
**Pure BN**	<0.1	N/A	N/A	N/A	N/A	N/A	98.6

* All temperature values have an error bar within ±0.5 °C; Mass remaining % data is obtained from TG analysis.
